# Epigenome-wide gene–age interaction study reveals reversed effects of *MORN1* DNA methylation on survival between young and elderly oral squamous cell carcinoma patients

**DOI:** 10.3389/fonc.2022.941731

**Published:** 2022-07-28

**Authors:** Ziang Xu, Yan Gu, Jiajin Chen, Xinlei Chen, Yunjie Song, Juanjuan Fan, Xinyu Ji, Yanyan Li, Wei Zhang, Ruyang Zhang

**Affiliations:** ^1^Jiangsu Key Laboratory of Oral Diseases, Nanjing Medical University, Nanjing, China; ^2^Department of Oral Special Consultation, Affiliated Stomatological Hospital of Nanjing Medical University, Nanjing, China; ^3^Department of Orthodontics, Affiliated Stomatological Hospital of Nanjing Medical University, Nanjing, China; ^4^Jiangsu Province Engineering Research Center of Stomatological Translational Medicine, Nanjing Medical University, Nanjing, China; ^5^Department of Biostatistics, Center for Global Health, School of Public Health, Nanjing Medical University, Nanjing, China; ^6^China International Cooperation Center for Environment and Human Health, Nanjing Medical University, Nanjing, China

**Keywords:** DNA methylation, age, gene–age interaction analysis, OSCC, overall survival

## Abstract

DNA methylation serves as a reversible and prognostic biomarker for oral squamous cell carcinoma (OSCC) patients. It is unclear whether the effect of DNA methylation on OSCC overall survival varies with age. As a result, we performed a two-phase gene–age interaction study of OSCC prognosis on an epigenome-wide scale using the Cox proportional hazards model. We identified one CpG probe, cg11676291*_MORN1_*, whose effect was significantly modified by age (HR_discovery_ = 1.018, *p* = 4.07 × 10^−07^, FDR-*q* = 3.67 × 10^−02^; HR_validation_ = 1.058, *p* = 8.09 × 10^−03^; *HR*_combined_ = 1.019, *p* = 7.36 × 10^−10^). Moreover, there was an antagonistic interaction between hypomethylation of cg11676291*_MORN1_* and age (HR_interaction_ = 0.284; 95% CI, 0.135–0.597; *p* = 9.04 × 10^−04^). The prognosis of OSCC patients was well discriminated by the prognostic score incorporating cg11676291*_MORN1_*–age interaction (*HR*_high vs. low_ = 3.66, 95% CI: 2.40–5.60, *p* = 1.93 × 10^−09^). By adding 24 significant gene–age interactions using a looser criterion, we significantly improved the area under the receiver operating characteristic curve (AUC) of the model at 3- and 5-year prognostic prediction (AUC_3-year_ = 0.80, AUC_5-year_ = 0.79, C-index = 0.75). Our study identified a significant interaction between cg11676291*_MORN1_* and age on OSCC survival, providing a potential therapeutic target for OSCC patients.

## Introduction

Oral squamous cell carcinoma (OSCC) is the most common subtype of head and neck malignancies as well as the most prevalent oral cancer worldwide (1), with an estimated 377,713 new cases and 177,757 deaths in 2020 (2). Despite recent breakthroughs in diagnosis and therapy, the prognosis of OSCC is still poor, with a 5-year survival rate of approximately 50% (3). As a complex disease, the progression of OSCC may be driven by a complex association pattern between genetic and environmental factors, i.e., gene–environment interaction (4).

DNA methylation is a reversible epigenetic modification without changing the DNA sequence (5). Nevertheless, its aberrant alterations play a decisive role in the occurrence and progression of various cancers (6, 7), including OSCC (8). Emerging evidence has demonstrated that DNA methylation may potentially serve as a prognostic biomarker of OSCC and a target for improved therapy (9, 10). However, the majority of these previous studies merely focused on identifying DNA methylation with marginal effect but overlooked gene–environment interaction. Age is a well-recognized environmental risk factor for the progression of many cancers (11), including OSCC (12, 13). Our previous gene–age interaction study of lung cancer revealed the reversed effects of *PRODH* DNA methylation on survival between young and elderly patients (14). Anyway, whether the effect of DNA methylation on OSCC survival varies with age remains largely unclear.

As a result, we hypothesized that there could be a gene–age interaction associated with OSCC survival at the DNA methylation level, and the age-specific epigenetic signatures could be more precise for therapeutic target discovery and prognostic prediction accuracy. Thus, we performed a two-phase epigenome-wide gene–age interaction study using subjects in The Cancer Genome Atlas (TCGA) as the discovery phase and subjects in the Gene Expression Omnibus (GEO) as the validation phase to identify age-specific, prognostic epigenetic biomarkers. A series of downstream analyses, i.e., sensitivity analysis, methylation–transcription analysis, gene network analysis, and immune cell composition analysis, were also conducted to explore the potential functions of the identified biomarkers.

## Methods

### Study populations

The level-3 TCGA-HNSCC DNA methylation data were downloaded from the UCSC XENA browser. Only samples whose tumors occurred in the oral cavity, tongue, floor of the mouth, buccal mucosa, hard palate, alveolar ridge, or lip were included in the discovery phase. In the validation phase, we retrieved and obtained OSCC patients’ clinical and DNA methylation data from the GEO (GSE75537) for further analysis.

### Quality control process for DNA methylation data

DNA methylation was assessed by the Illumina Infinium Human Methylation 450 Array. We used the R package *CHAMP* to process level-3 data from TCGA and the GEO. Ineligible CpG probes were removed if they met any of the quality control (QC) criteria: (i) non-CpG probes, (ii) common SNPs located in the position of the CpG probe or 10 bp flanking regions, (iii) cross-reactive probes, (iv) sex chromosome probes, (v) deletion rates >20%, and (vi) failed QC in either TCGA or GEO cohorts. Types I and II probe corrections were normalized using BMIQ normalization. They were further adjusted for batch effects (ComBat function in R package *sva*) according to the best pipeline by a comparative study (15). **Supplementary Figure S1** describes the details of the QC process. Subjects with no overall survival time were also removed. Finally, 372 subjects (**Table 1**) and 361,060 CpG probes remained in the subsequent association analysis.

**Table 1:** Demographic and clinical descriptions of subjects in the discovery phase (TCGA), the validation phase (GEO), and the combined dataset, respectively.

Characteristic	TCGA (*N* = 319)	GEO (*N* = 53)	Combined (*N* = 372)
Age (years)	61.76 ± 13.15	49.36 ± 13.47	59.99 ± 13.87
Gender (*N* (%))			
Male	212 (66.5)	42 (79.3)	254 (68.3)
Female	107 (33.5)	11 (20.7)	118 (31.7)
Smoking status (*N* (%))			
Never	89 (28.7)	–	89 (28.7)
Former	125 (40.3)	–	125 (40.3)
Current	96 (31.0)	–	96 (31.0)
Unknown	9	53	62
T stage (*N* (%))			
T1	19 (6.0)	13 (24.5)	32 (8.7)
T2	100 (31.6)	15 (28.3)	115 (31.2)
T3	79 (25.0)	12 (22.7)	91 (24.7)
T4	113 (35.8)	13 (24.5)	126 (34.1)
T*x*	5 (1.6)	0 (0)	5 (1.3)
Unknown	3	0	3
N stage (*N* (%))			
N0	165 (52.2)	25 (47.2)	190 (51.5)
N1	57 (18.0)	8 (15.1)	65 (17.6)
N2	83 (26.3)	20 (37.7)	103 (27.9)
N3	2 (0.6)	0 (0)	2 (0.5)
N*x*	9 (2.9)	0 (0)	9 (2.5)
Unknown	3	0	3
M stage (*N* (%))			
M0	302 (95.6)	45 (84.9)	347 (94.0)
M1	2 (0.6)	0 (0)	2 (0.5)
M*x*	12 (3.8)	8 (15.1)	20 (5.5)
Unknown	3	0	3
Clinical stage (*N* (%))			
Early (I–II)	88 (28.3)	17 (34.0)	105 (29.1)
Late (III–IV)	223 (71.7)	33 (66.0)	256 (70.9)
Unknown	8	3	11
Race (*N* (%))			
White	276 (89.3)	–	276 (89.3)
Other	33 (10.7)	–	33 (10.7)
Unknown	10	53	63
Survival months			
Mean (95% CI)	95.0 (93.8–96.3)	71.2 (60.5–81.8)	91.6 (89.6–93.7)
Death (%)	148 (46.4)	15 (28.3)	163 (43.8)

Restricted mean survival time is provided because the median was not available.

### Study populations and gene expression data

In TCGA cohort, 307 OSCC patients had complete mRNA sequencing data. TCGA mRNA sequencing data processing and quality control were performed by TCGA working group. Level-3 mRNA expression data were downloaded from the UCSC XENA database and further checked for quality. The expression value of each gene was transformed on a log_2_ scale before association analysis.

### Statistical analysis

#### A two-phase gene–age interaction study

**Figure 1:**
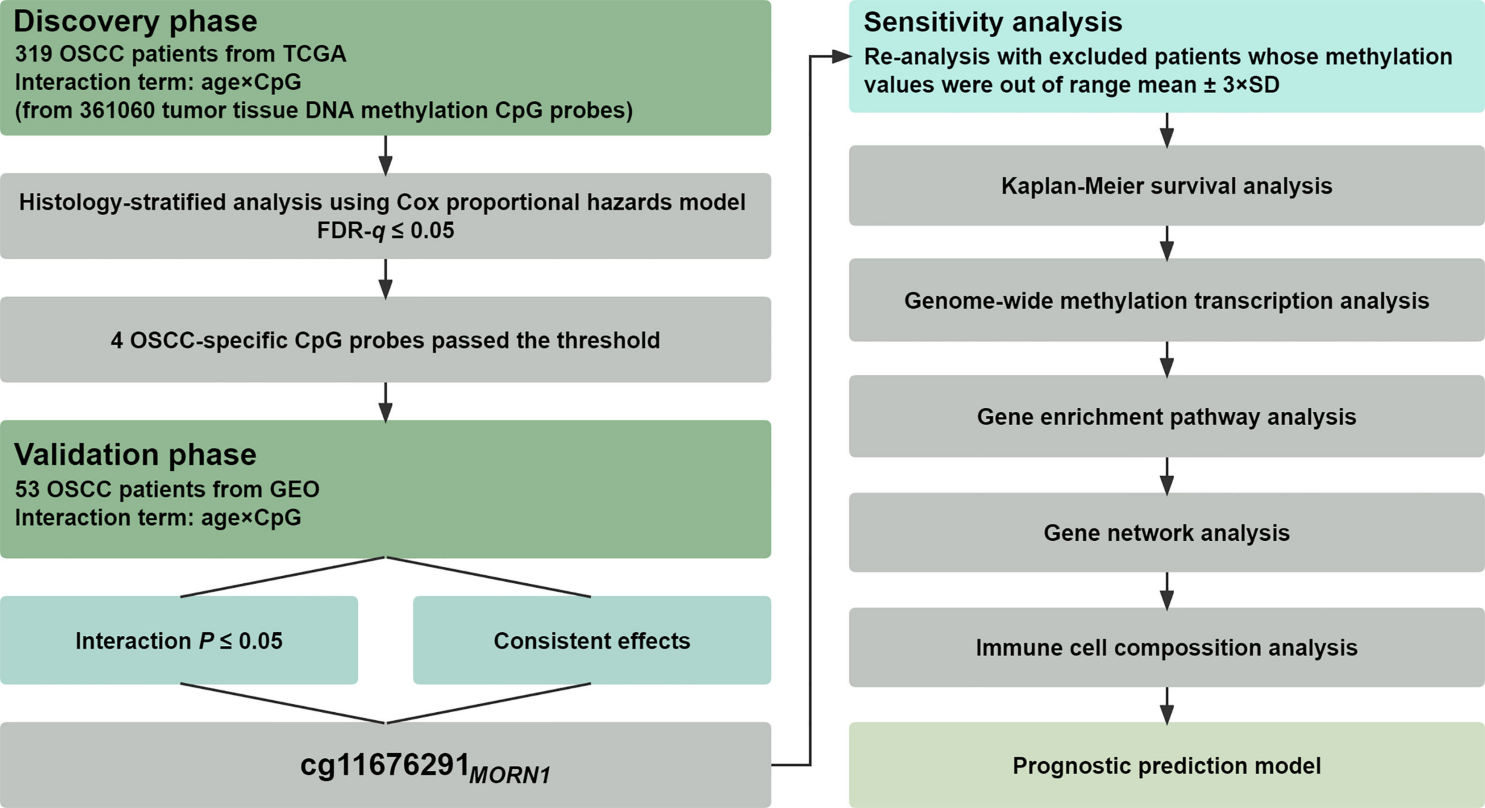
Flow chart of study design and statistical analyses. Patients from TCGA were used in the discovery phase for biomarker screening, whereas patients from the GEO were used for biomarker validation.

The statistical analysis pipeline was depicted in **Figure 1**, showing a two-phase study to examine gene–age interactions associated with OSCC overall survival on the epigenome-wide scale. In the discovery phase, the interaction between DNA methylation and age on overall survival was tested in the TCGA cohort using a histology-stratified Cox proportional hazards model adjusted for age, smoking status, gender, and TNM stage. Hazard ratios (HRs) and 95% confidence intervals (CIs) were calculated for incremental methylation per 1% level. Multiple test corrections were performed by controlling the false discovery rate (FDR) at the 5% level, and further replications were performed in the validation phase. Significant probes were finally retained if they met all the following criteria: (i) FDR-*q* ≤ 0.05 in the discovery phase; (ii) *p* ≤ 0.05 in the validation phase; and (iii) consistent effect direction across two phases. Patients were excluded if their methylation values were out of range to mean ± 3 × standard deviations (SD) in the sensitivity analysis. Kaplan–Meier survival curves were used to describe the difference in survival between hypomethylated and hypermethylated patients.

#### Functional analysis of CpG probes with significant interactions

Potential genes *trans*-regulated by epigenetic biomarkers in TCGA were identified by genome-wide methylation–transcription correlation analysis using a linear regression model adjusted for the same covariates aforementioned. Functional annotation and gene enrichment pathway analysis (FDR-*q* ≤ 0.05) of the Kyoto Encyclopedia of Genes and Genomes (KEGG) and Gene Ontology (GO) for potential *trans*-regulated genes were performed using the R Package *WebGestaltR*. Furthermore, these genes associated with overall survival were selected for gene network analysis using the Cytoscape application plugin *GeneMANIA* (16). Gene hubs which highly connected to nodes in the module were defined as those having the highest connectivity. To explore the difference in tumor immune cell subtypes among subgroups, we quantified the composition of 22 tumor-infiltrating immune cells (TIICs) using CIBERSORT, a linear support vector regression-based deconvolution algorithm (17).

#### Development of a prognostic prediction model

By using a looser criterion (FDR-*q* ≤ 0.10 in the discovery phase; *p* ≤ 0.05 in the validation phase), more gene–age interactions were further selected and incorporated into a prognostic prediction model of OSCC. The accuracy of prediction was represented using the time-dependent receiver operating characteristic (ROC) curve and was measured by the area under the ROC curve (AUC) using the R package *survivalROC*. The 95% CI and *p-*value for AUC increments were calculated from 1,000 bootstrap samples. The concordance index (C-index), an average accuracy of predictive survival across follow-up years, was also calculated to estimate predictive performance.

In order to illustrate the different DNA methylation effects on survival in populations of different ages, we used two classification criteria to define young and elderly patients: (1) the UN standard age of 65 as the threshold (18), (2) the boundary of 95% CI (BoCI) threshold calculated based on the HR of CpG probe. Furthermore, continuous variables were summarized as mean ± standard deviation (SD), while categorized variables were described by frequency (*n*) and proportion (%) in description analysis. All statistical analyses were performed in R version 4.0.3 (The R Foundation for Statistical Computing, Vienna, Austria).

## Results

### A significant gene–age interaction was identified in the two-phase study

In the discovery phase, four gene–age interactions were identified with FDR-*q* ≤ 0.05, of which only one remained significant (*p* ≤ 0.05) in the validation phase and showed a more robust association in the combined data (**Supplementary Table S1**). The CpG probe, cg11676291*_MORN1_*, located in the *MORN* Repeat Containing 1 (*MORN1*) (**Supplementary Table S2**), together with age, showed a significant interaction effect on OSCC survival (HR_interaction_ = 1.018, 95% CI: 1.011–1.025, *p* = 4.07 × 10^−07^, FDR-*q* = 3.67 × 10^−02^ in the discovery phase; HR_interaction_ = 1.058, 95% CI: 1.015–1.103, *p* = 8.09 × 10^−03^ in the validation phase; HR_interaction_ = 1.019, 95% CI: 1.013–1.025, *p* = 7.36 × 10^−10^ in the combined data). Furthermore, in the sensitivity analysis, by removing outliers in the methylation data, the significant interaction effect was again confirmed in the two-phase study (**Supplementary Table S3**). Stratified analyses by gender, TNM stage, and smoking status showed no significant heterogeneity among those subgroups. Meanwhile, the association between cg11676291*_MORN1_*–age interaction and overall survival remained significant in all subgroups (**Supplementary Figure S2**), except for the current smoker subgroup with a very limited sample size (*n* < 100).

**Figure 2:**
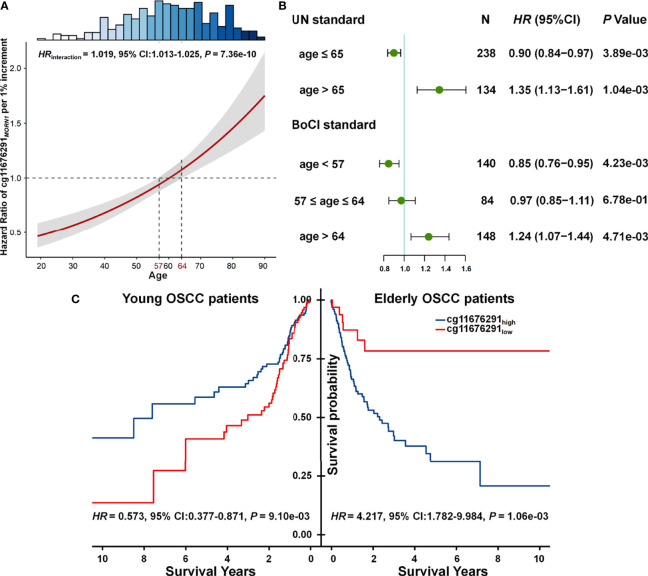
Gene–age interaction on survival of OSCC patients. **(A)** *HR* of cg11676291*_MORN1_* 1% per increment of methylation level among differently aged patients. The 95% CI bands of *HRs* for patients aged <57 and >64 years were significantly different. The top histogram shows the distribution of age. **(B)** Forest plots of *HR* of cg11676291*_MORN1_* 1% per increment of methylation level in young and elderly OSCC patients, categorized based on BoCI and UN standards. **(C)** Kaplan–Meier survival curves of low and high DNA methylation groups among young and elderly OSCC patients were defined using the BoCI standard.

Statistical interaction between two factors can be defined as a phenomenon where the effect of one factor is modified by another one (19). Combined with our results, we observed that the effect of cg11676291*_MORN1_* was modified by age, where the CpG probe changed from a protective factor for OSCC survival in young patients to a risk factor in elderly patients (**Figure 2A**). Thus, age was obviously a modifier of the association between cg11676291*_MORN1_* and overall survival. By categorizing patients into young and elderly groups according to UN criteria (≤65 vs. >65 years) or BoCI boundaries (<57 vs. >64 years) in the combined data, both stable results showed the reversed effects of cg11676291*_MORN1_* between two age subgroups (**Supplementary Table S4**). Hypermethylation of cg11676291*_MORN1_* favored survival in young OSCC patients (HR_UN_ = 0.900; 95% CI: 0.838–0.967; *p* = 3.89 × 10^−03^; HR_BoCI_ = 0.849; 95% CI: 0.760–0.950; *p* = 4.23 × 10^−03^) but was not conducive for survival in elderly OSCC patients (HR_UN_ = 1.345; 95% CI: 1.127–1.605; *p* = 1.04 × 10^−03^; HR_BoCI_ = 1.240; 95% CI: 1.068–1.440; *p* = 4.71 × 10^−03^) (**Figure 2B**). Based on the optimal cutoff value of cg11676291*_MORN1_*, Kaplan–Meier curves also confirmed the reversed effects across two age groups (HR_high vs. low_ = 0.573; 95% CI: 0.377–0.871; *p* = 9.10 × 10^−03^ in young OSCC patients; HR_high vs. low_ = 4.217; 95% CI: 1.782–9.984; *p* = 1.06×10^-03^ in elderly OSCC patients) based on BoCI criteria (**Figure 2C**). All these results indicated that young OSCC patients with hypermethylation of cg11676291*_MORN1_* had better survival, while the conclusion only held for the elderly OSCC patients with hypomethylation of cg11676291*_MORN1_*.

In addition, we also assessed the interaction pattern of cg11676291*_MORN1_* methylation level (low vs. high) and age (young vs. elderly) on OSCC survival using the group with the highest survival rate (young patients with cg11676291*_MORN1_* hypermethylation) as a reference (**Supplementary Table S5**). The main effect of cg11676291*_MORN1_* hypomethylation was HR = 1.629 (95% CI: 0.935–2.839), and the main effect of advanced age was HR = 2.461 (95% CI: 1.463–4.138). However, their joint effect was HR = 1.138 (95% CI: 0.635–2.042), which was less than the product of the two main effects (1.629 × 2.461 = 4.009), indicating there was an antagonistic interaction between cg11676291*_MORN1_* hypomethylation and advanced age (*HR*_interaction_ = 0.284; 95% CI: 0.135–0.597; *p* = 9.04 × 10^−04^).

### Genome-wide *trans*-regulation analyses of cg11676291*_MORN1_*

**Figure 3:**
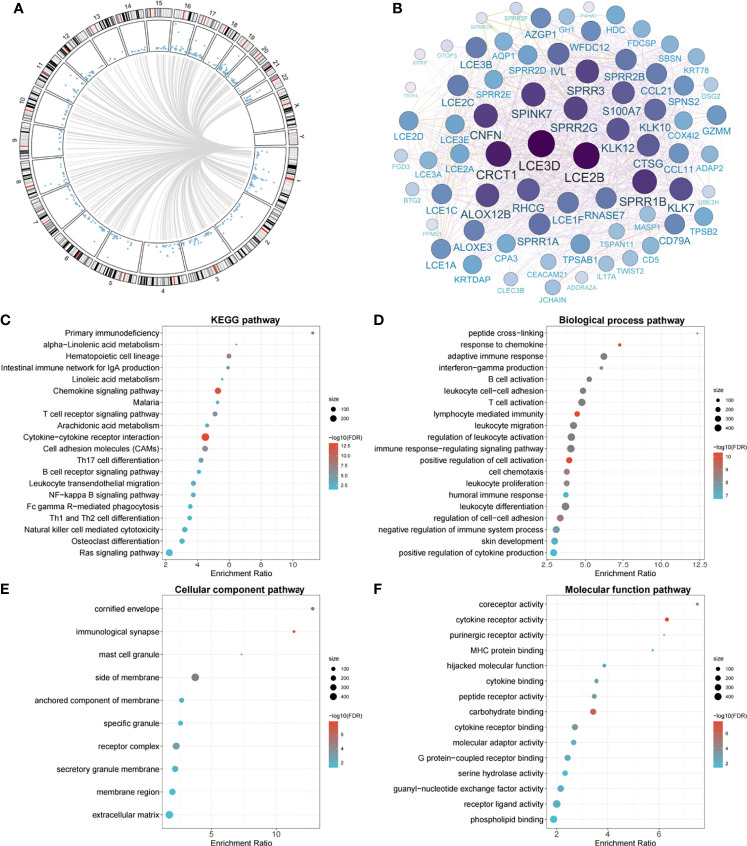
Circos plot of genome-wide methylation–transcription analysis, gene network of prognostic genes *trans*-regulated by cg11676291*_MORN1_*, and significant pathways of gene enrichment pathway analysis. **(A)** Circos plot of genes *trans*-regulated by cg11676291*_MORN1_* in the TCGA cohort. Blue points ordered by genomic position represent *P* values derived from linear regression between gene expression and cg11676291*_MORN1_*. Grey lines represent significant correlations with FDR-*q ≤*0.05. **(B)** The gene network plot of 50 genes *trans*-regulated by cg11676291*_MORN1_* and associated with OSCC overall survival. The size represents the connectivity degree of each node. **(C)** The top 20 significant KEGG pathways. **(D)** The top 20 significant biological process pathways. **(E)** The top 10 significant cellular component pathways. **(F)** The top 15 significant molecular function pathways.

Genome-wide methylation–transcription analysis by the linear regression model indicated that the expressions of 586 genes were significantly *trans*-regulated by cg11676291*_MORN1_* (**Figure 3A**). Among them, 50 genes were further significantly associated with OSCC overall survival, which were evaluated by the Cox proportional hazards model adjusted for the same covariates aforementioned. The gene network identified two hub genes (*LCE3D* and *LCE2B*) with the highest degree of connectivity (**Figure 3B**). Meanwhile, these epigenetically *trans*-regulated genes were significantly enriched in 22 KEGG pathways (**Figure 3C**), including several cancer-related pathways. In addition, GO enrichment analysis identified 71 biological process pathways (**Figure 3D**), 10 cellular component pathways (**Figure 3E**), and 16 molecular functional pathways (**Figure 3F**). Moreover, *MORN1* expression was significantly (*P_p_*_= 0,_ *_q_*_= 1_ = 1.88 × 10^−02^ and *P_p_*_= 1,_ *_q_*_= 1_ = 2.75 × 10^−02^) associated with OSCC overall survival as shown by Kaplan–Meier survival curves **Supplementary Figure S3** which was confirmed by the Harrington–Fleming test that was designed for the late or delayed effect of the variable during the follow-up (20).

### Gene–Age Interaction-Empowered Prognostic Prediction Model

**Figure 4:**
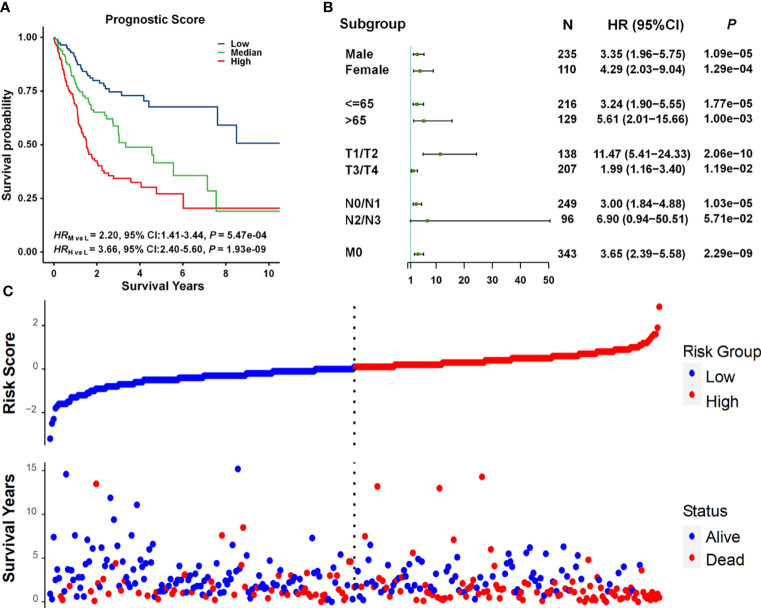
Survival analysis of prognostic scores. **(A)** Kaplan–Meier survival curves for patients grouped by prognostic scores. Patients were categorized into three subgroups by using the tertiles of prognostic scores. The number of patients in each group was 115. **(B)** Forest plots of results from association analysis of the relationship between prognostic scores and overall survival. *HR*, 95% CI, and *p-*values were derived from the Cox proportional hazards regression model. **(C)** The relationship between prognostic scores and survival status.

We developed a prognostic prediction model incorporating cg11676291*_MORN1_*–age interaction and clinical information. All patients in the combined dataset were categorized into low-, middle-, and high-risk groups by the tertile of the prognostic score, which was a weighted linear combination of all variables in the model. Compared to the low-risk group, the mortality risk was 2.20 and 3.66 times higher in the middle- and high-risk groups, respectively (*HR*_medium vs. low_ = 2.20, 95% CI = 1.41–3.44, *p* = 5.47 × 10^−04^; HR_high vs. low_ = 3.66, 95% CI = 2.40–5.60, *p* = 1.93 × 10^−09^) (**Figure 4A**). The prognostic score was significantly associated with overall survival in almost all subgroups (**Figure 4B**), except for the N2/N3 subgroup exhibiting a boundary significance (*p* = 5.71 × 10^−02^) with a limited sample size (*n* < 100). Also, the risk score was correlated with survival status. As displayed in **Figure 4C**, we observed more deaths in these patients with high-risk scores.

**Figure 5:**
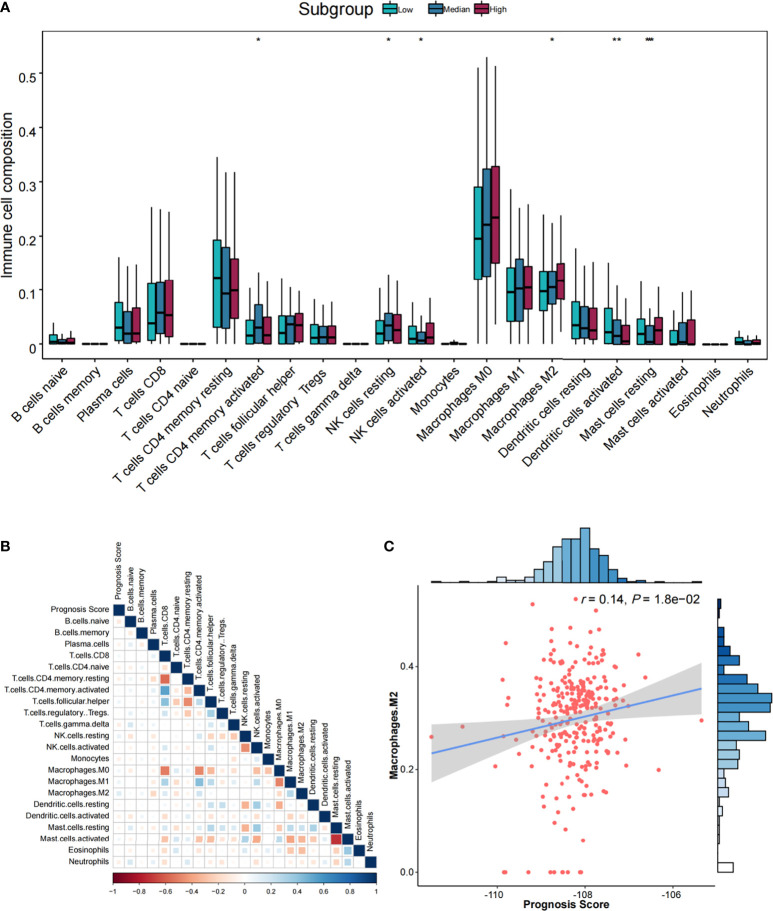
The association analysis between immune cells and prognostic score. **(A)** Comparisons of the abundances of 22 immune cells in three risk groups. ^*^p < 0.05, ^**^p < 0.01, and ^***^p < 0.001. **(B)** Heatmap of correlations among immune cells and prognostic score. Correlation coefficients were derived from Pearson correlation analysis. **(C)** Scatter plot and association analysis between prognostic score and M2 macrophages.

Furthermore, six types of TIICs were significantly and differently distributed among low-, medium-, and high-risk groups (**Figure 5A**), including CD4 memory resting T cells, NK cells resting, activated NK cells, M2 macrophages, dendritic cells activated, and resting mast cells. By Pearson correlation analysis of 22 TIICs and prognostic score (**Figure 5B**), only M2 macrophages exhibited a significant positive correlation (*r* = 0.14, *p* = 1.80 × 10^−02^) (**Figure 5C**).

**Figure 6:**
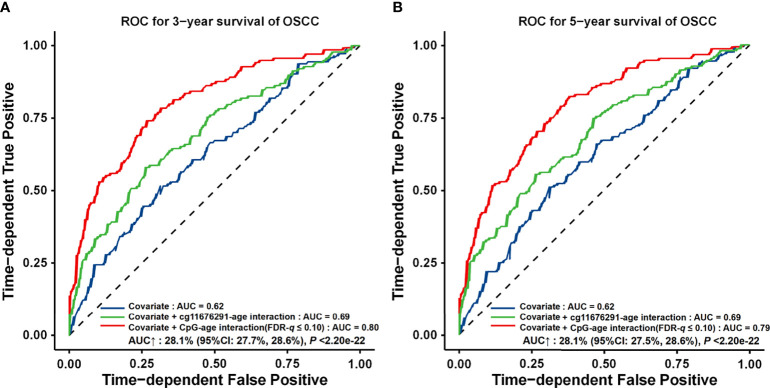
ROC curves for different prognostic prediction models using clinical information, gene–age interactions with FDR‐*q* ≤ 0.05 or FDR‐*q* ≤ 0.10. **(A)** Three‐year survival prediction. **(B)** Five‐year survival prediction. The AUC increase (%) was evaluated by comparing the model with gene–age interactions and the model with only the covariates. p-values and 95% CIs were calculated by using 1,000 bootstrap samples and z tests.

Compared to the model with only demographic and clinical variables (AUC_3 years_ = 0.62, AUC_5 years_ = 0.62, and C-index = 0.61), the interaction-empowered prognostic prediction model had a slightly improved accuracy by adding the cg11676291*_MORN1_*–age interaction (AUC_3 years_ = 0.69, 11.2% increase; AUC_5 years_ = 0.69, 12.1% increase; and C-index = 0.66, 9.0% increase). Furthermore, by adding 24 gene–age interactions obtained using a looser criterion, the AUC increased by 28.1% (95% CI: 27.7%–28.6%, *p* < 2.20 × 10^−16^) and 28.1% (95% CI: 27.5%–28.6%, *p* < 2.20 × 10^−16^) for 3-year and 5-year survival, respectively (AUC_3 years_ = 0.80, AUC_5 years_ = 0.79, and C-index = 0.75) ( **Figure  6**).

## Discussion

This is the first attempt to study the interaction effect between DNA methylation and age on OSCC overall survival on an epigenome-wide scale. In this two-phase study, we systematically investigated gene–age interactions and identified one CpG probe, cg11676291*_MORN1_*, whose effect on survival varied with age. Also, there was an antagonistic interaction between hypomethylation and advanced age. Meanwhile, these genes *trans*-regulated by cg11676291*_MORN1_* were significantly associated with a series of immune pathways and immune cells. Finally, the gene–age interaction empowered the prognostic prediction model of OSCC and possessed a better capability to predict patients’ overall survival.

Accumulating evidence indicated that gene–gene and gene–environment interactions play important roles in the occurrence, progression, and prognosis of various complex diseases (21, 22), especially cancers (23–26). Our study found that the effect of DNA methylation on OSCC survival may change with age, indicating gene–age interactions might be potentially involved in OSCC prognosis. Furthermore, the gene–age interaction might boost the prediction accuracy and lead to satisfactory performance of 3- and 5-year survival predictions for OSCC, which was in accordance with our previous studies of lung cancer (27, 28). Therefore, complex association patterns among multiple factors should also be factored in for the OSCC study.

Moreover, we observed that *MORN1* expression was also associated with OSCC survival. *MORN1* is a protein-coding gene associated with sacral defects with anterior meningocele (29). Interestingly, *MORN1* has been shown to be involved in budding (30), cell division (31), and epidermal formation of *Toxoplasma gondii* (32). Chronic infection of *T. gondii*, an opportunistic parasitic disease, affects a quarter of the world’s population (33). *T. gondii* achieves persistence in host cells by manipulating many signaling pathways, which are closely related to immune and inflammatory responses (34), and may cause severe damage to immunodeficient or immunocompromised hosts. Epidemiology in various region surveys has shown that the seroprevalence of *T. gondii* is significantly increased in both elderly patients and cancer patients (35, 36). Moreover, the other genes associated with cg11676291*_MORN1_* were also enriched in immune-related pathways, including the T-cell receptor signaling pathway, B-cell receptor signaling pathway, Th17 cell differentiation, and Th1 and Th2 cell differentiation. Therefore, we speculated that the altered effect of cg11676291*_MORN1_* might be caused by *T. gondii* infection because of decreased immunity in aging OSCC patients. However, further biological experiments exclusively designed for the *MORN1*–age interaction are warranted.

Furthermore, two hub genes (*LCE3D* and *LCE2B*) in the gene network have also been confirmed as prognostic biomarkers of laryngeal squamous cell carcinoma (LSCC) (37). Since there may be no anatomical heterogeneity between LSCC and OSCC, these two genes may share the same mechanisms in the progression of head and neck squamous cell carcinoma.

Our study has several strengths. First, to our knowledge, this may be the first study to investigate the interaction between DNA methylation and age on OSCC survival on an epigenome-wide scale, which provided new insights into the prognosis of OSCC patients at different ages. Second, to improve the robustness of the interaction signal, we adopted a two-phase study design (discovery phase vs. validation phase), FDR correction of multiple tests, and sensitivity analysis to control the false positives. Third, the interaction pattern between cg11676291*_MORN1_* and age was visually illustrated using interaction and forest plots. Finally, our prognostic model incorporating DNA methylation–age interactions could help physicians make clinical decisions.

We also acknowledge some limitations. First, we only performed gene–age interaction in the current study, and interactions between DNA methylation and other clinical variables are expected in future studies. Second, statistical power may be limited due to the small size (*n* = 53) and the high censored rate (71.7%) of the GEO cohort. Nevertheless, the interaction between cg11676291*_MORN1_* and age was still significant in such a scenario, indicating its robustness. Third, the gene–age interaction-empowered prognostic prediction model requires DNA methylation information, which potentially increases the cost of clinical testing. Nevertheless, we envision low-cost and high-efficiency tests in the future will facilitate the application of our proposed model. Finally, since the majority of the population of the TCGA cohort is Caucasian (89.3%), the generalization of our results to other ethnicities should be cautioned.

## Conclusion

We identified one CpG probe (cg11676291) located in *MORN1*, together with age, which had a genome-wide significant gene–age interaction effect on OSCC survival. The effect of cg11676291*_MORN1_* on survival was modified by age, indicating that OSCC survival was driven by a complex association pattern.

## Data availability statement

The original contributions presented in the study are included in the article/**supplementary material**. Further inquiries can be directed to the corresponding authors.

## Author contributions

ZX, YG, WZ, and RZ contributed to the study design. ZX, YG, JC, XC, YS, JF, XJ, and YL contributed to data collection, statistical analysis, and interpretation. ZX, YG, JC, WZ, and RZ drafted the manuscript. All authors contributed to the critical revision of the manuscript and approved its final version. Financial support and study supervision were provided by WZ and RZ.

## Funding

This study was funded by the Natural Science Foundation of Jiangsu Province (BK20191354 to RZ and SBK2017043261 to WZ) and the Priority Academic Program Development of Jiangsu Higher Education Institutions (PAPD). RZ was partially supported by the Qing Lan Project of the Higher Education Institutions of Jiangsu Province and the Outstanding Young Level Academic Leadership Training Program of Nanjing Medical University.

## Conflict of interest

The authors declare that the research was conducted in the absence of any commercial or financial relationships that could be construed as a potential conflict of interest.

## Acknowledgments

The authors thank TCGA and the GEO for contributing clinical, DNA methylation, and RNA sequencing data, as well as all study subjects who participated in the two study cohorts.

## Publisher’s note

All claims expressed in this article are solely those of the authors and do not necessarily represent those of their affiliated organizations, or those of the publisher, the editors and the reviewers. Any product that may be evaluated in this article, or claim that may be made by its manufacturer, is not guaranteed or endorsed by the publisher.

## Abbreviations

OSCC, oral squamous cell carcinoma; TCGA, The Cancer Genome Atlas; GEO, Gene Expression Omnibus; QC, quality control; SNP, single-nucleotide polymorphisms; BMIQ, Beta-Mixture Quantile; HR, hazard ratio; CI, confidence interval; FDR, false discovery rate; SD, standard deviation; KEGG, Kyoto Encyclopedia of Genes and Genomes; GO, Gene Ontology; TIICs, tumor-infiltrating immune cells; ROC, receiver operating characteristic; AUC, area under the receiver operating characteristic curve; C-Index, concordance index; BoCI, boundary of 95% CI.
