# Supplementary material for: Epigenome-wide gene–age interaction study reveals reversed effects of MORN1 DNA methylation on survival between young and elderly oral squamous cell carcinoma patients
Source: Front Oncol. 2022 Jul 28;12:941731. doi: 10.3389/fonc.2022.941731 (PMC9366171; doi:10.3389/fonc.2022.941731)
Supplement: Supplementary file 1 [file DataSheet_1.docx]

Supplementary files

**Table S1**. Results for four gene-age interactions identified from a two-phase epigenome-wide association study of OSCC overall survival.

| CpG | TCGA | | | | | GEO | | | | Combined | | | |
| --- | --- | --- | --- | --- | --- | --- | --- | --- | --- | --- | --- | --- | --- |
|  | *HR* | 95% CI | | *P* | FDR-*q* | *HR* | 95% CI | | *P* | *HR* | 95% CI | | *P* |
| cg11676291 | 0.349 | 0.233 | 0.524 | 5.34×10^-07^ | - | 0.063 | 0.011 | 0.374 | 8.39×10^-03^ | 0.321 | 0.225 | 0.458 | 3.34×10^-10^ |
| Age | 0.187 | 0.097 | 0.360 | 3.52×10^-07^ | - | 0.004 | 6.43×10^-05^ | 0.242 | 2.35×10^-03^ | 0.164 | 0.092 | 0.292 | 9.99×10^-10^ |
| Interaction | 1.018 | 1.011 | 1.025 | 4.07×10^-07^ | 3.67×10^-2^ | 1.058 | 1.015 | 1.103 | 8.09×10^-03^ | 1.019 | 1.013 | 1.025 | 7.36×10^-10^ |
| cg10602135 | 0.064 | 0.022 | 0.187 | 5.57×10^-07^ | - | 0.158 | 0.017 | 1.503 | 1.08×10^-01^ | 0.073 | 0.030 | 0.174 | 4.43×10^-09^ |
| Age | 0.012 | 0.002 | 0.065 | 1.97×10^-07^ | - | 0.079 | 0.001 | 5.451 | 2.40×10^-01^ | 0.016 | 0.004 | 0.062 | 2.85×10^-09^ |
| Interaction | 1.046 | 1.029 | 1.064 | 1.81×10^-07^ | 2.18×10^-2^ | 1.026 | 0.983 | 1.072 | 2.40×10^-01^ | 1.044 | 1.029 | 1.058 | 2.58×10^-09^ |
| cg18342703 | 0.289 | 0.199 | 0.421 | 8.53×10^-11^ | - | 0.109 | 0.012 | 0.954 | 4.53×10^-02^ | 0.310 | 0.223 | 0.431 | 3.24×10^-12^ |
| Age | 0.153 | 0.080 | 0.294 | 1.57×10^-08^ | - | 0.020 | 3.21×10^-4^ | 1.262 | 6.44×10^-02^ | 0.170 | 0.195 | 0.305 | 2.61×10^-09^ |
| Interaction | 1.020 | 1.013 | 1.027 | 1.22×10^-08^ | 2.51×10^-3^ | 1.041 | 0.998 | 1.086 | 6.43×10^-02^ | 1.019 | 1.012 | 1.025 | 1.97×10^-09^ |
| cg18585512 | 0.430 | 0.322 | 0.575 | 1.20×10^-08^ | - | 0.637 | 0.352 | 1.153 | 1.36×10^-01^ | 0.514 | 0.410 | 0.644 | 7.46×10^-09^ |
| Age | 0.295 | 0.192 | 0.453 | 2.40×10^-08^ | - | 0.422 | 0.124 | 1.440 | 4.81×10^-02^ | 0.376 | 0.269 | 0.527 | 1.31×10^-08^ |
| Interaction | 1.014 | 1.009 | 1.018 | 1.39×10^-08^ | 2.51×10^-3^ | 1.009 | 0.996 | 1.023 | 1.67×10^-01^ | 1.011 | 1.007 | 1.015 | 7.92×10^-09^ |

**Table S2**. Annotation information for the significant CpG probe identified by the epigenome-wide gene-age interaction study of OSCC overall survival.

| CpG probe | Gene | CHR | Region | CpG islands | Relation to CpG islands |
| --- | --- | --- | --- | --- | --- |
| cg11676291 | *MORN1* | 1 | Body | chr1:2322481-2323764 | Body-shelf |

**Table S3**. Results of the gene-age interaction in sensitivity analysis.

| CpG | TCGA | | | | GEO | | | | Combined | | | |
| --- | --- | --- | --- | --- | --- | --- | --- | --- | --- | --- | --- | --- |
|  | *HR* | 95% CI | | *P* | *HR* | 95% CI | | *P* | *HR* | 95% CI | | *P* |
| cg11676291 | 0.344 | 0.208 | 0.569 | 3.15×10^-05^ | 0.063 | 0.011 | 0.374 | 8.39×10^-03^ | 0.306 | 0.200 | 0.468 | 4.85×10^-08^ |
| Age | 0.167 | 0.074 | 0.377 | 1.67×10^-05^ | 0.004 | 6.43×10^-05^ | 0.242 | 2.35×10^-03^ | 0.141 | 0.070 | 0.284 | 3.78×10^-08^ |
| Interaction | 1.019 | 1.010 | 1.028 | 1.34×10^-05^ | 1.058 | 1.015 | 1.103 | 8.09×10^-03^ | 1.021 | 1.013 | 1.028 | 2.93×10^-08^ |

In sensitivity analysis, patients were excluded if their methylation values were out of range mean ± 3 × standard deviation on logit_2_ transformed scale.

Table S4. Results of cg11676291*_MORN1_* methylation effect on OSCC survival in young and elderly populations defined using UN and BoCI standards in the combined data.

| Standard | Subpopulation | *HR* | 95% CI | | *P* |
| --- | --- | --- | --- | --- | --- |
| UN | Young (age ≤65 years) | 0.900 | 0.838 | 0.967 | 3.89×10^-03^ |
|  | Elderly (age >65 years) | 1.345 | 1.127 | 1.605 | 1.04×10^-03^ |
| BoCI | Young (age <57 years) | 0.849 | 0.760 | 0.950 | 4.23×10^-03^ |
|  | Elderly (age >64 years) | 1.240 | 1.068 | 1.440 | 4.71×10^-03^ |

**Table S5.** Joint and interaction effects of low methylation and elderly age on the prognosis of OSCC.

| Effect type | Low methylation ^a^ | Elderly ^a^ | Number | Death | Crude mortality | *HR* (95% CI) ^b^ | *P* |
| --- | --- | --- | --- | --- | --- | --- | --- |
|  | No | No | 64 | 22 | 34.38% | Reference |  |
| Main effect _1_ | Yes | No | 65 | 31 | 47.69% | 1.629 (0.935,2.839) | 8.49×10^-02^ |
| Main effect _2_ | No | Yes | 74 | 46 | 62.16% | 2.461 (1.463,4.138) | 6.85×10^-04^ |
| Joint effect | Yes | Yes | 69 | 25 | 36.23% | 1.138 (0.635,2.042) | 6.64×10^-01^ |
| Interaction ^c^ |  |  |  |  |  | 0.284 (0.135,0.597) | 9.04×10^-04^ |

^a^ Patients were divided into two groups (low and high) based on the median of cg11676291*_MORN1_* methylation levels. Age classification criteria were based on 95% confidence interval (CI) criteria boundaries (young: <57 years; elderly: >64 years).

^b^ The main effects of hypomethylation and old age, and their joint and interaction effcts were derived from Cox proportional hazards models.

^c^ Interaction = joint effect ÷ (main effect _1_ × main effect _2_), which was calculated as: 0.284 ≈ 1.138 ÷ (2.461 × 1.629).

**Figure S1.** Quality control processes for DNA methylation data.


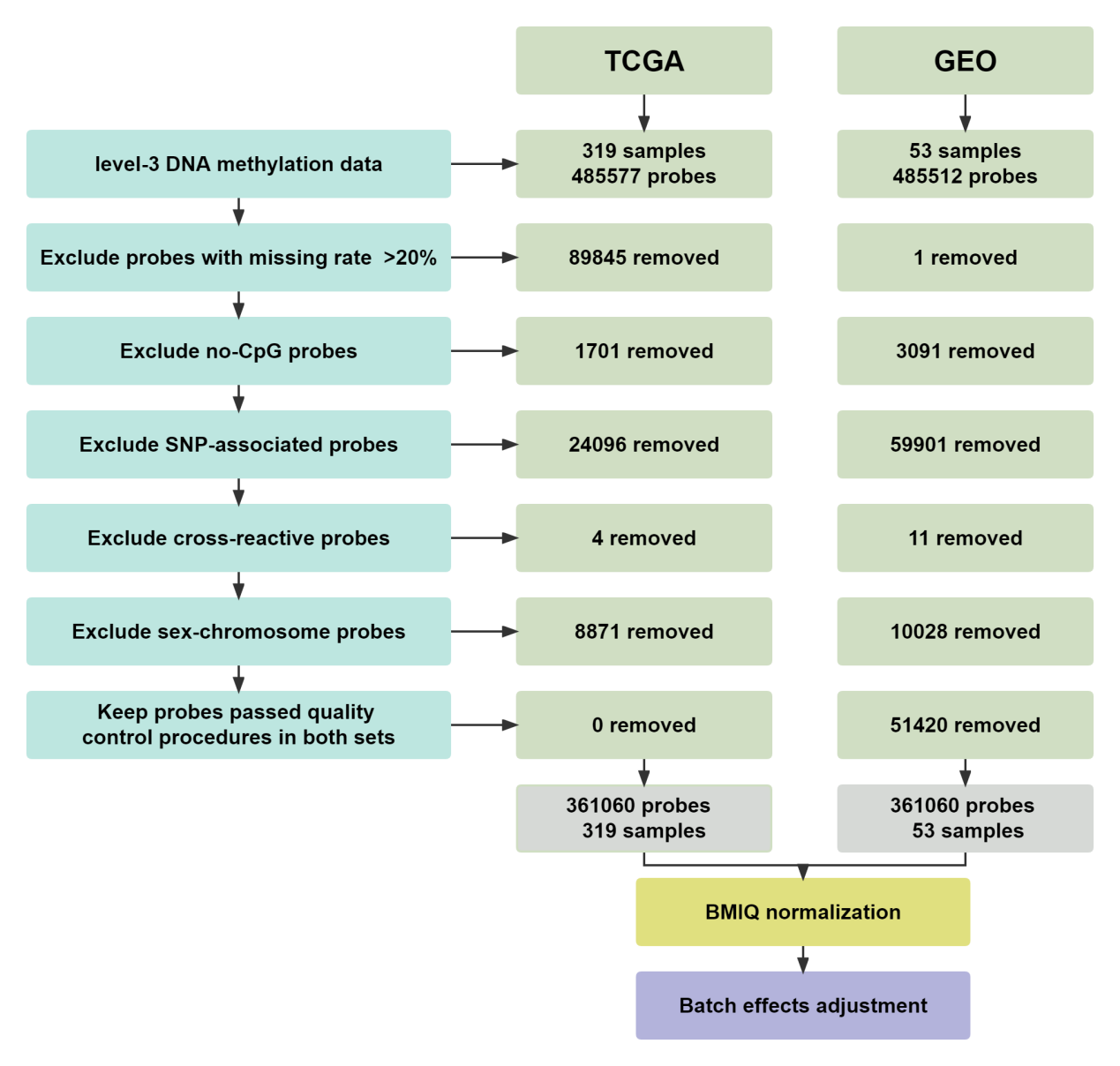


Figure S2. Results of the cg11676291*_MORN1_*-age interaction effect in subgroups categorized by covariates.


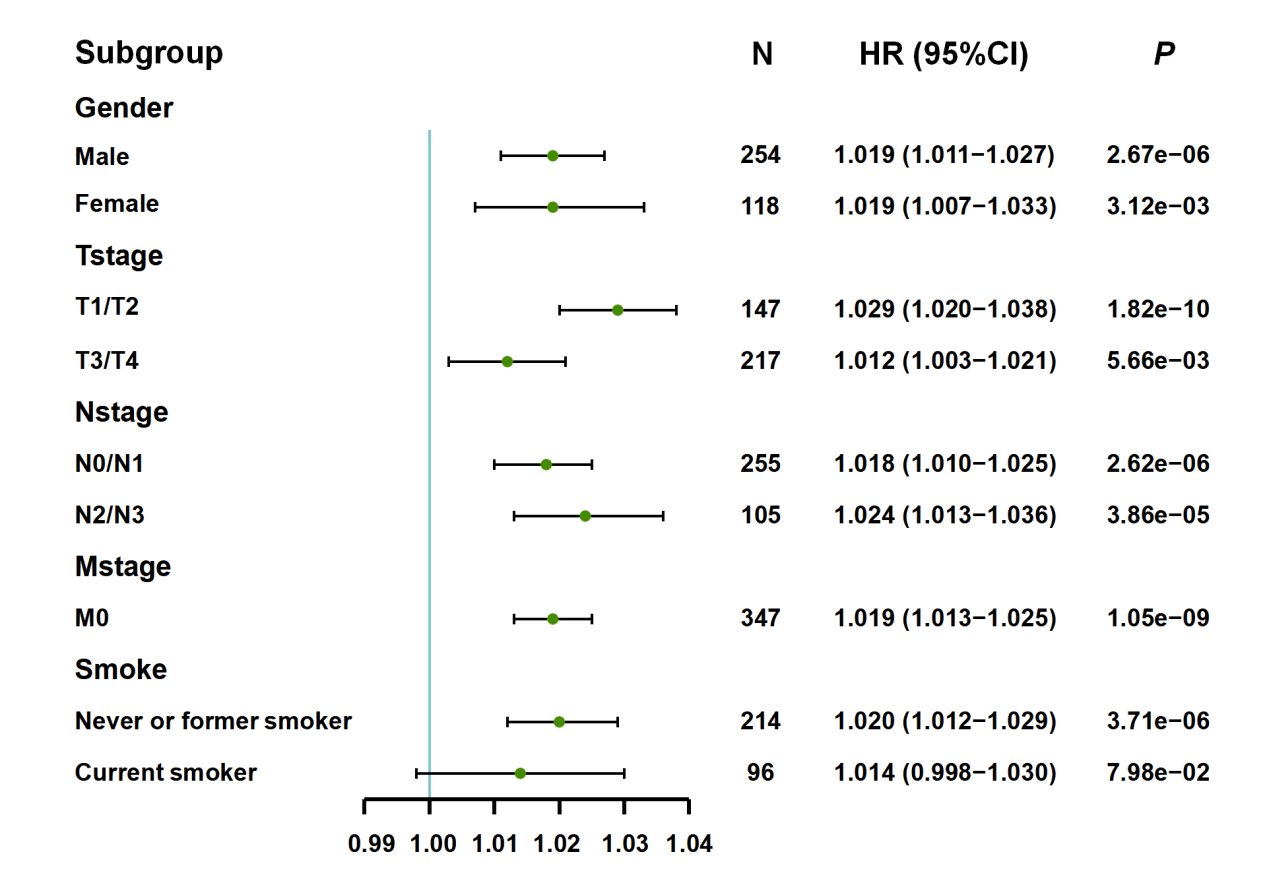


Figure S3. Kaplan-Meier survival curves for OSCC patients with low and high *MORN1* expressions.


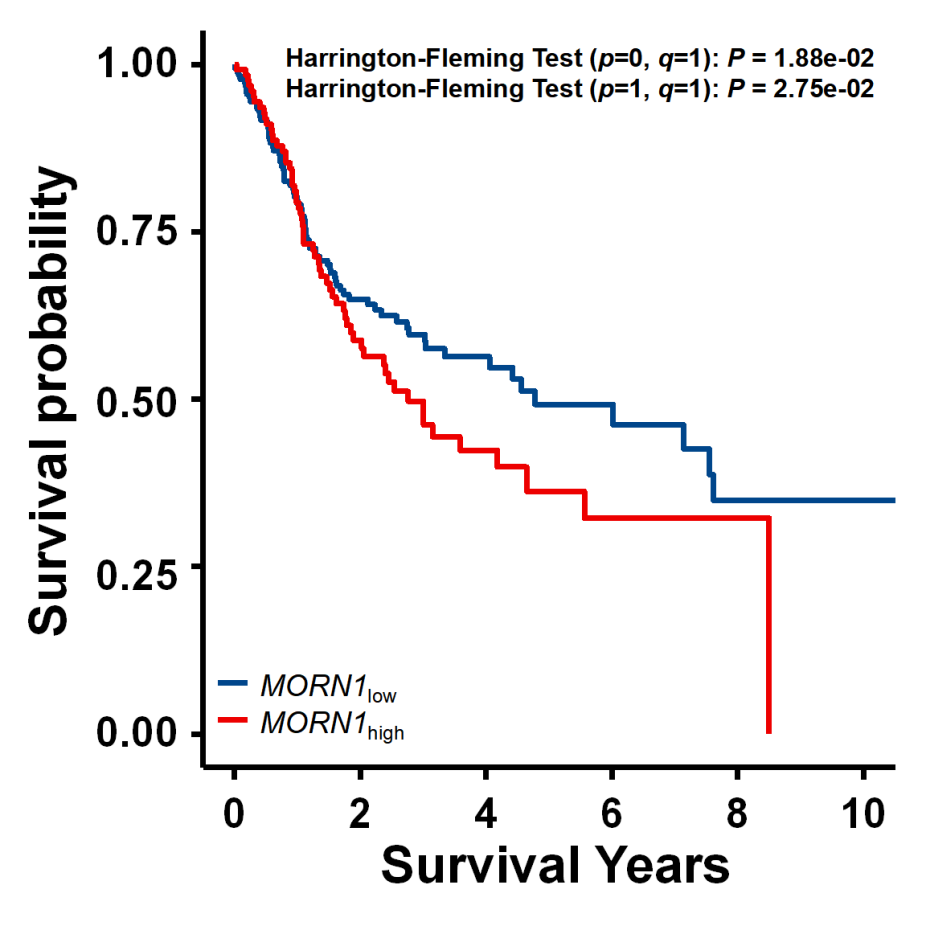


*P* values were derived from Harrington-Fleming test with parameters: (*p* = 0 , *q* =1) and (*p* = 1 , *q* =1). The former one was designed for late effects of variable during the follow-up and the later one was designed for both early and late effects of variable during the follow-up.
